# Differential Diagnosis and Interdisciplinary Workup of a Pediatric Patient With an Unknown Immune Condition: Chronic Respiratory Distress Secondary to Viral Illness and Developmental Consequences

**DOI:** 10.7759/cureus.53109

**Published:** 2024-01-28

**Authors:** Camryn Daidone, Sheyenne Carper

**Affiliations:** 1 Research, Edward Via College of Osteopathic Medicine, Shreveport, USA; 2 Pediatrics, Willis-Knighton Health System, Shreveport, USA

**Keywords:** hyper-ige syndrome, wiskott-aldrich syndrome, gastroesophageal reflux disease (gerd), pediatric asthma, child behaviour and development, respiratory care, interdisciplinary collaboration, delayed developmental milestones

## Abstract

We present a case of a three-year-old African American male, born at term, who initially presented with bronchiolitis at six months and has since experienced recurrent episodes of respiratory distress and hospitalizations. The patient also has severe eczema, developmental delays, and recurrent viral illnesses. Despite thorough evaluations from various specialists, such as pulmonology, allergy, and gastroenterology, the underlying cause remained elusive. The differential diagnosis for this case is as follows: severe persistent asthma with a possible link to genetic mutations such as CDHR3, hyper-IgE syndrome, atypical presentation of Wiskott-Aldrich syndrome, and severe gastroesophageal reflux disease (GERD) with aspiration pneumonitis. This patient’s chronic condition has contributed to several developmental consequences, including failure to gain weight and possible hypoxic encephalopathy, leading to delays in cognitive and motor milestones and speech delays. Aggressive medical management, especially long-term systemic steroids, raises concerns about future complications. Through this case, we highlight the importance of thorough workups and an interdisciplinary approach to diagnosing and managing an unknown immune condition, as well as consistent pediatric primary care follow-up to assess development and coordinate necessary support. Here, we aim to address a gap in research on the unique presentations of pediatric respiratory distress symptoms by formulating a comprehensive differential diagnosis and exploring the various ways that chronic respiratory illness can contribute to developmental deficits such as speech and cognitive delays in pediatric patients. This study calls for further research into genetic contributions to asthma, diverse presentations of GERD, prevention of viral illnesses, alternative treatments minimizing steroid use, and an understanding of the impact of chronic respiratory distress on cognitive and language development in children. Thorough workups and interdisciplinary approaches are essential for effective diagnosis and management.

## Introduction

In approximately the first six months of life, full-term infants have an improved ability to fight off illnesses due to maternal passive immunity. Infants who are born premature or who have gestational complications, such as placental insufficiency, may experience immunodeficiency shortly after birth [1]. After this window for neonatal passive immunity ends, children who are genetically predisposed to have primary immunodeficiency may begin to show symptoms, including severe infections. Children with hyper-IgE spectrum disorders may begin to exhibit eczema, chronic bacterial infections of the lungs and skin, and staph abscesses [2]. Patients with Wiskott-Aldrich syndrome (WAS) begin to show a triad of severe infections, eczema, and increased bleeding due to thrombocytopenia [3,4]. For children predisposed to asthma, environmental triggers may cause episodes of respiratory distress and other symptoms of atopy such as eczema, allergic rhinitis, and allergies [5]. This list includes only a few of the hundreds of subsets of primary immunodeficiency diseases that impact approximately 500,000 people in the United States. Some subsets of primary immunodeficiency are tested in neonatal (newborn) screening, such as severe combined immunodeficiency (SCID), while many are not.

Regardless of which immune condition is present, chronic illnesses and aggressive treatments can alter the trajectory of normal child development. Children with immunodeficiencies and chronic diseases are more likely to experience developmental delays. For example, patients with chronic diseases, such as cystic fibrosis and chronic kidney disease, are much more likely to experience malnutrition and failure to thrive due to the increased metabolic demand [6]. For patients who experience hypoxia early in childhood due to birth complications or other pathologies, there is an increased risk of cognitive disability and delays in the development of attention, executive function, memory, language, and sometimes motor skills [7]. In addition to chronic illness impacting child development, treatment of pediatric health conditions with medications such as long-term systemic steroids can lead to chronic health complications as well [8].

Here, we present a case of a three-year-old male whose symptoms began with a case of bronchiolitis at six months of age and progressed to recurrent episodes of respiratory distress necessitating aggressive medical management and multiple hospitalizations. This patient’s symptoms included recurrent viral illnesses, severe eczema, and developmental delays. This study presents an interdisciplinary workup of the patient’s case and a working differential diagnosis and evaluates the impact of this patient’s condition on his development. Through this case, we highlight the importance of thorough workups and a multidisciplinary approach to diagnosing and managing an unknown immune condition, as well as consistent pediatric primary care follow-up to assess development and coordinate necessary support. Here, we aim to address a gap in research on the unique presentations of pediatric respiratory disease by formulating a comprehensive differential diagnosis and exploring the various ways that chronic respiratory illness can contribute to developmental deficits such as speech and cognitive delays in pediatric patients.

## Case presentation

An African American male carried to term (39 weeks, 0 days) first presented to the emergency department at six months of age with acute bronchiolitis due to an unspecified organism. The patient was stabilized and sent home with a course of systemic steroids. This would be the first episode of an ongoing battle of respiratory illnesses and dermatitis, requiring numerous hospitalizations in the years to follow.

At birth, he presented with Apgar scores of 8 and 9 at one and five minutes, respectively. The birth was induced due to a nuchal cord but otherwise was free of complications. The patient's mother had exposure to hepatitis C, but otherwise, there was no known exposure to perinatal infections or teratogens. The patient received all recommended newborn vaccinations, and the newborn physical examination was normal, including cardiopulmonary and neurological assessments. The patient initially failed his newborn hearing screen, but after two confirmatory audiology evaluations performed in the first 20 months of life, the patient demonstrated normal hearing, and it was determined that the newborn screening results were invalid. The patient’s newborn screening was negative for all tested conditions, such as congenital adrenal hyperplasia, amino acid disorders, SCID, cystic fibrosis, phenylketonuria, and galactosemia. This patient has a family history of diabetes mellitus, anemia, allergies, and eczema in first-degree relatives.

The patient most recently presented to his pediatrician for a well-child visit at three years old and had already presented to numerous emergency departments while being hospitalized for respiratory symptoms on several occasions.

Hospitalizations and critical care

Each hospitalization followed a similar sequelae of acute-onset respiratory distress necessitating breathing treatments. The most minor of the episodes only required oxygen supplementation, bronchodilator treatments, and observation, while other exacerbations required several-day hospital admissions for pneumonia where the patient required mechanical ventilation.

**Figure 1:**
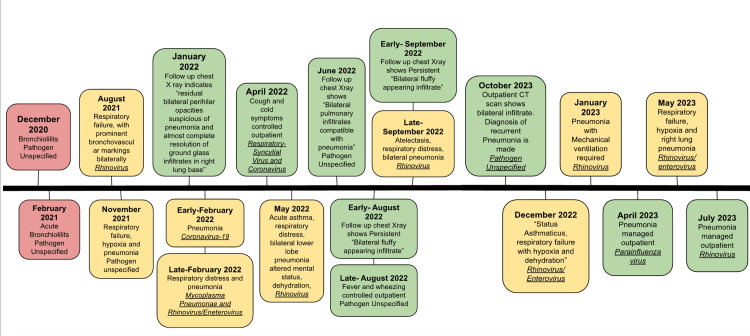
A timeline showing the patient's admissions to the emergency department (red), hospital admissions (yellow), and outpatient management of illnesses/follow-up visits (green) from December 2020 to July 2023. The timeline indicates the month of presentation, diagnosis/indication for treatment, and the associated pathogen (if known)

These respiratory illnesses typically presented with chest X-ray (CXR) findings indicative of bilateral lower lobe pneumonia (most commonly worse in the right lower lung base) or diffuse opacities. Though these episodes of respiratory infection were not always attributed to a specific organism, some pathogens known to cause respiratory infections in this patient were rhinovirus/enterovirus, coronavirus, respiratory syncytial virus, COVID-19, mycoplasma pneumoniae, and parainfluenza virus. The most common pathogen discovered during these exacerbations was rhinovirus. Several hospital discharge notes indicated dehydration, significant hypoxia, and altered mental status during the course of treatment. Figure 1 shows a timeline of the patient's presentations and diagnoses and indicates which pathogens are responsible if known. "Respiratory distress" and "respiratory failure" are often used as diagnoses for hospital admissions, but their definitions are provider-dependent. Presentations that specifically met the criteria for hypoxia are indicated.

Pulmonology

Starting at 14 months of age, this patient was referred to pulmonology for close evaluation and management of respiratory symptoms. He was originally diagnosed with moderate persistent reactive airway disease (RAD) and was prescribed fluticasone propionate inhalation aerosol (Flovent, PRASCO LLC) and albuterol for control of his symptoms. Throughout pulmonology workups, it was noted that the patient’s symptoms were aggravated by viral illnesses and lying down, and that symptoms worsened in the late night and early morning. He had been prescribed aggressive albuterol treatments as well as inhaled corticosteroids and systemic steroids as needed. He was treated with step-up therapy using the following medications: budesonide, montelukast (Singulair, Merck), budesonide (Pulmicort, AstraZeneca), and fluticasone propionate inhalation aerosol (Flovent, PRASCO LLC), all of which appeared to control his symptoms until they were exacerbated by a viral illness requiring hospital admission. This patient’s diagnosis has since changed from moderate RAD to severe persistent asthma.

When the patient was 26 months of age, left hilar fullness was noted on a CXR during a diagnosis of pneumonia with *Mycoplasma pneumoniae* and rhinovirus/enterovirus. The patient’s pulmonologist wanted to rule out a bronchogenic cyst and sent him for a follow-up chest computerized tomography (CT) scan. On this CT, no hilar fullness was noted, establishing a bronchogenic cyst as unlikely and further supporting the diagnosis of recurrent bilateral pneumonia. Outside of this incidence of a possible bronchogenic cyst, the patient was never reported as having cysts, abscesses, or strictures in the lungs or respiratory tract.

A bronchoscopy was also performed at 28 months of age, which displayed lipid-laden macrophages after bronchoalveolar lavage (BAL) was performed.

Allergy/immunology

This patient began seeing an allergist at 16 months of age. At that time, it was discovered that he had severely elevated IgE in response to cat and dog dander, eggs, and peanuts. A full allergy workup found that IgG (including four subclasses), IgA, and IgM values were within normal limits while IgE values were increased (483 kU/L, reference range: ≤97.0). This workup also identified peripheral eosinophilia. Complete blood counts performed during this patient’s hospitalizations and specialist workups were consistently within normal limits, with the exception of elevated calcium, a high platelet count, and an occasional increased red blood cell count.

Gastroenterology

With the initial concern of aspiration pneumonitis, this patient was referred to gastroenterology at 29 months of age. After multiple swallow studies showing normal results, it was concluded that it was unlikely that aspiration pneumonitis was the primary cause of this patient's symptoms. However, this patient was found to have profound gastroesophageal reflux disease (GERD) and slow weight gain, both of which were managed using prescribed proton-pump inhibitors (PPIs). After a trial of PPIs, the patient’s mother reported that the patient had an improved appetite, decreased GERD symptoms such as coughing after eating and food aversion, and weight gain.

An esophagogastroduodenoscopy performed at 33 months of age indicated a small hiatal hernia with normal histopathology of the stomach and esophagus. There was no evidence of esophagitis or eosinophilia reported.

Pediatric primary care

Pediatric well-child visits are at the heart of this patient’s medical management. Starting at 10 months of age, this patient presented with severe atopic dermatitis (eczema) and respiratory symptoms. This patient’s eczema was consistently managed through lifestyle modifications, skin hygiene, and topical corticosteroids. At 10 months of age, this patient was reported as meeting all physical, language, and social/emotional developmental milestones expected for his age.

During a well-child check at 19 months of age, macrocephaly was noted on the physical exam, and the patient began to fall behind in verbal and cognitive developmental milestones. At that time, he began speech therapy for speech delays. With speech therapy, the patient began to improve, but by three years of age, his speech therapists still reported a significant discrepancy between receptive and expressive language. At three years old, this patient was able to comprehend spoken language but struggled with language expression.

Initially, this patient failed his newborn hearing screen and was referred to audiology for further evaluation. Initial audiological evaluations at 20 months indicated poor tympanic membrane mobility and potential hearing loss, but by 22 months of age and with a second opinion from an ear, nose, and throat doctor, it was confirmed that this patient had no hearing deficits. In subsequent speech therapy evaluations, there was no indication that this patient struggled with hearing.

Failure to gain weight has been a consistent concern for this patient. He was at the 16.9 percentile at 10 months of age, 14.8% at 12 months, 2.7% at 16 months, 4.1% at 18 months, and 3.4% at two years of age. After medical management, including the use of PPIs to manage GERD and control of respiratory symptoms, his weight improved from 3.4 percentile at two years to the 41st percentile by three years of age.

At the patient’s three-year-old well-child visit, he failed to meet expected motor milestones for his age group, such as toilet training, standing on one foot, and dressing himself. Shortly after this visit, the patient presented for follow-up after a hospitalization during which the patient was supported with a ventilator for one week due to pneumonia, respiratory failure, and acidosis. At the time of discharge, an MRI scan of his brain displayed suspicion of diffuse axonal injury secondary to hypoxic encephalopathy. The patient was scheduled for follow-up brain imaging, but at the time that this case report was written, no further imaging had been performed.

## Discussion

This case of a three-year-old with a currently unknown immune condition highlights the importance of thorough evaluation and multidisciplinary management of this condition and close pediatric follow-up to assess child development. Below, we have highlighted the differential diagnosis for his condition.

Differential diagnosis

Severe Persistent Asthma

This patient’s presentation of recurrent episodes of wheezing and respiratory distress in the setting of viral illnesses, predominantly rhinovirus, eczema, allergy, peripheral eosinophilia, and increased IgE strongly suggest a diagnosis of asthma with increased Th2 atopy and eosinophilia. The etiology of asthma follows a two-hit hypothesis, where patients are predisposed via genetic mutations, and then the onset of symptoms is triggered by environmental stressors [5]. In this case, it is likely that the patient has a mutation of a gene, such as a mutation of 17q21 (which causes dysregulation of the ICAM1 receptor eosinophil production and is associated with virus-induced wheezing and eosinophilia in children) or the CDHR3 gene (which contains a rhinovirus-c receptor and is associated with recurrent acute hospitalizations following rhinovirus infections prior to six years of age) [5]. It is important to note that asthma is a multifactorial disease, and it is very likely that this patient's presentation of severe persistent asthma could be a result of a variety of genetic abnormalities as well as environmental triggers such as pathogens, allergens, or even exercise. While this patient may be a candidate for further investigation into CDHR3 gene testing, it is likely that the etiology of this patient's asthma is complex and requires an individualized treatment plan.

In patients with asthma, type 2 inflammatory responses such as IgE hypersensitivity and overactivation of eosinophils, basophils, and mast cells are triggered by an environmental stressor such as an allergen, viral illness, or smoke exposure, which triggers a cascade of atopic events leading to eczema, asthma, and allergic rhinitis. For patients under the age of one year with non-atopic asthma, this environmental trigger is likely to be illness from respiratory syncytial virus, while rhinovirus is a likely causative pathogen in patients over the age of one year with atopic asthma [5].

There is evidence suggesting that viral illnesses play a large role in asthma exacerbations and that rhinovirus is a common pathogen to trigger severe asthma exacerbations due to its significant antigenic diversity, the triggered Th2-based inflammatory response, decreased interferon responses, and disrupted airway-epithelial barrier in patients with asthma [5]. Additionally, modifications to the CDHR3 gene have been linked to asthma and contain a rhinovirus-C receptor, leaving patients particularly susceptible to rhinovirus-induced asthma exacerbations [5].

For patients with severe persistent asthma prone to exacerbations by viral illness, prevention of viral illness is imperative in the prevention of exacerbations. Monoclonal antibodies such as palivizumab developed to prevent respiratory syncytial virus may be considered, and these patients may benefit from yearly influenza vaccines [5]. Due to the antigenic diversity of rhinovirus, there is currently no good treatment or prevention for this virus, but as rhinovirus is proving to be a strong inciting factor for asthma exacerbations in patients with severe asthma, future research should strive to develop improved methods for the prevention and management of rhinovirus [5,9]. As for the management of asthma exacerbations, systemic steroids are still the first-line treatment, despite the potential consequences of prolonged steroid administration.

The patient in this case may benefit from testing for mutations in genes such as CDHR3 and 17q21 and the administration of systemic steroids and potentially anti-IgE monoclonal antibodies such as omalizumab, which have shown promise in this population through improving their anti-viral responses [5].

Hyper-IgE Syndrome

This patient’s elevated IgE levels and history of recurrent respiratory infections such as pneumonia and dermatitis certainly place hyper-IgE syndrome on the differential diagnosis list. Hyper-IgE syndrome is a primary immunodeficiency that begins with the onset of symptoms at approximately six months of age. Hyper-IgE syndrome has been classified as a spectrum of diseases that includes a subset that is primarily based on atopy. The etiology of these conditions is complex and poorly understood and warrants further investigation, but evidence suggests it may be attributed to genetic factors such as mutations on the zinc finger protein impacting STAT3 transcription [2]. Patients with this condition traditionally present with eczema, pneumonia, and staph abscesses in the skin and lungs. Typically, the pathogens causing infection in patients with hyper-IgE syndrome are bacterial and most often include *Staphylococcus aureus*, *Streptococcus pneumoniae*, and *Haemophilus influenzae* [10]. While it is certainly possible that this patient could have a primary immunodeficiency on the spectrum of hyper-IgE syndromes, the lack of abscesses and the fact that the etiology of most of his pneumonia and infections is viral and not bacterial suggest that hyper-IgE syndrome may not be the primary differential diagnosis for this patient.

Due to the complex pathogenesis of hyper-IgE syndromes, the clinical presentation may vary drastically between patients. It is possible to have hyper-IgE syndrome without the presence of abscesses, and it has been of particular importance in this patient that regular CXRs are performed to rule out any potential abscesses. One CXR noted the possible presence of a bronchogenic cyst, warranting further investigation. Bronchogenic cysts are most commonly found in children and contain respiratory epithelium, hyaline cartilage, and bronchogenic glands [11]. These cysts can be found in the respiratory tree or subcutaneous tissue, and there have been cases where bronchogenic cysts coincide with lung abscesses [12]. Ultimately, this patient was found to not have any evidence of bronchogenic cysts or lung abscesses after evaluation with a chest CT. Additionally, periodic IgE testing, monitoring for progression of skin and lung abscesses, and a potential trial of anti-IgE medications such as omalizumab may be helpful in further assessing for hyper-IgE syndrome.

Wiskott-Aldrich Syndrome

WAS is an X-linked genetic disorder where mutations in the WAS gene and WAS protein impact the development and differentiation of hematopoietic stem cells into blood and immune cells [3]. This disorder is characterized by severe infection, thrombocytopenia, increased bleeding, eczema, and ultimately an increased risk of future malignancy [3]. As this is an X-linked disorder, WAS disproportionately affects males. This patient is a male with eczema and increased infections, so WAS is certainly a possible diagnosis. However, this patient has no history of bleeding symptoms and no history of thrombocytopenia. In fact, this patient has a history of occasional elevated platelet values.

In a case report of a patient with a classical presentation of a known WAS gene mutation, this patient had reports of recurrent sinus infections, skin abscesses, and an eczematous rash starting at day nine of life, along with an elevated IgE value and microthrombocytes on a blood smear [4]. While it is possible that the patient in this study has asymptomatic hematologic complications, the diagnosis of WAS is less likely. A genetic evaluation of the WAS gene may be helpful in this patient, as a diagnosis of WAS would make this patient a good candidate for hematopoietic stem cell transplantation [3].

Severe GERD and Aspiration Pneumonitis

Another possible diagnosis for this patient is severe GERD and aspiration pneumonitis. This patient has a known history of GERD and a hiatal hernia found on endoscopy. A study published in 2020 highlights a potential bidirectional relationship between GERD and asthma in children [13]. Reflux can destroy the respiratory-epithelial barrier and increase the presence of asthma symptoms and susceptibility to infection. On the other hand, asthma can cause systemic inflammation and disruption of the vagus nerve, which in turn weakens the respiratory diaphragm and leads to the development of reflux [13]. There is also an association between GERD and asthma flare-ups that may be stronger in pediatric patients [14]. In children, weakening of the lower esophageal sphincter and chronic GERD can lead to hiatal hernias. Hiatal hernias can also be congenital due to malformation of the diaphragm [15].

For this patient, the presence of lipid-laden macrophages in BAL may be suggestive of microaspiration. Lipid-laden macrophages are a nonspecific finding that can be suggestive of obstruction in cases such as endogenous lipoid pneumonia, pulmonary alveolar proteinosis, or fat embolisms in sickle cell disease [16]. In pediatric patients, lipid-laden macrophages are most commonly associated with aspiration and warrant further investigation, including a barium swallow study [16]. This patient’s barium swallow study was unremarkable for signs of aspiration, which indicates that GERD may not be the primary diagnosis behind this patient’s symptoms but likely plays a role in the exacerbation of his symptoms and may even make him more vulnerable to viral respiratory illnesses. Regardless, management of this patient’s GERD and hiatal hernia is likely vital to controlling his respiratory symptoms.

Implications of illness on child development

This patient’s chronic illness has certainly contributed to a delay in various developmental milestones. A primary issue for this patient was failure to gain weight. This is commonly seen in patients with chronic diseases due to increased metabolic demand [6]. Additionally, GERD has been linked to failure to thrive in infants and young children due to insufficient caloric intake and an impaired ability to swallow [17]. Pediatric patients with neurologic deficits may also be more likely to develop GERD [17]. Though this is typically associated with failure to thrive in infants, insufficient caloric intake and the “food fear” associated with this patient’s reflux are likely contributing to his failure to thrive. Upon management of hiatal hernia and reflux with aggressive proton pump inhibitor treatments, this patient began to gain weight. This highlights the importance of managing GERD in preventing additional developmental delay for this patient.

This patient has had progressive delays in cognitive and motor milestones, the most significant of which were impairments in language expression. Extensive speech evaluation has indicated that this patient’s delay in speech is expressive and not due to a language comprehension problem. Though originally this patient had a suspected impairment of hearing, the lack of delay in language comprehension makes it unlikely that hearing impairment is present or a root cause of this delay. More likely, this patient’s history of reflux may be contributing to speech impairment, as reflux pharyngitis can lead to inflammation, damage, and stricture in the larynx [18]. It is important to note that these developmental delays are unlikely to have been caused by a single factor and are likely the result of multiple physical and environmental conditions this patient has experienced.

Additionally, hypoxic encephalopathy is likely contributing to this patient’s developmental delay. Though most commonly studied in the context of neonatal hypoxic encephalopathy secondary to birth complications, there is a significant association between hypoxic/ischemic brain injury and neurodevelopmental deficits such as impaired cognitive development, memory and language delays, and sometimes functional motor deficits that persist even into late childhood and early adolescence [3,19]. This patient’s frequent episodes of respiratory distress have been linked to hypoxia and are likely to precede developmental challenges.

Finally, this patient has received aggressive medical management that may be linked to adverse developmental and medical outcomes. For example, the primary treatment for the management of severe asthma is systemic corticosteroids. Long-term systemic corticosteroid use in pediatric patients has been linked to chronic health complications such as diabetes, an increased risk of infection, osteoporosis, and psychiatric disorders [8]. The impacts of long-term systemic corticosteroids may not be present in this patient for several years, but this is certainly something to consider in the management of chronic asthma [8].

## Conclusions

This case study of a three-year-old with episodes of chronic respiratory distress, viral illness, and allergic symptoms highlights four differential diagnoses: severe asthma, WAS, hyper-IgE syndrome, and severe GERD with microaspiration. This paper is the first of its kind in evaluating a unique presentation of chronic pediatric respiratory distress and emphasizes a profound need for future research in genetic contributions to asthma, various presentations and implications of GERD, prevention of viral illnesses, alternative treatments to respiratory distress that minimize systemic steroid use, and the impact of chronic respiratory distress and hypoxia on child cognitive and language development. This case calls for the use of genetic testing in evaluating patients with similar respiratory symptoms. Testing for abnormalities in genes such as 17q21, CDHR3, and those involved in STAT3 transcription may be indicated, and whole-genome sequencing should be considered to identify possible genetic triggers that could help to formulate individualized treatment programs. Further, the evaluation of this patient’s difficulty in meeting developmental milestones emphasizes the importance of thorough workups and interdisciplinary management of pediatric patients with chronic health conditions.
